# Vitamin D Status and Test Utilization: A Five-Year Analysis

**DOI:** 10.7759/cureus.106542

**Published:** 2026-04-06

**Authors:** Salima Al Maqbali, Hoor Al Maharbi, Shatha Al Handhali, Thuriya Al Hashimi, Sanam Anwar, Tasnim Alswaii

**Affiliations:** 1 Pathology and Laboratory Medicine, Sohar Hospital, Sohar, OMN; 2 Family and Community Medicine, National University of Science and Technology (NU), Sohar, OMN; 3 Public Health and Epidemiology, National University of Science and Technology (NU), Sohar, OMN

**Keywords:** 25-hydroxyvitamin d, clinical indications, laboratory demand management, primary healthcare, test utilization, vitamin d, vitamin d deficiency, vitamin d insufficiency

## Abstract

Background

Vitamin D testing has increased markedly over the past decade, often exceeding evidence-based recommendations that discourage routine screening in low-risk populations. This growing demand has raised concerns regarding test overutilization, misalignment with clinical indications, and the uncertain impact on patient management. This study aimed to assess vitamin D status, clinical indications for testing, and associated comorbidities over a five-year period in North Batinah, Oman, while evaluating the clinical utility of vitamin D testing in routine practice.

Methodology

A retrospective cross-sectional study included all vitamin D requests by the primary healthcare institutes sent to Sohar Hospital, the sole regional laboratory providing serum 25-hydroxyvitamin D (25(OH)D) testing. All Omani and non-Omani residents who underwent testing between January 2018 and November 2022 were included. Vitamin D status was classified as deficient (<30 nmol/L), insufficient (30-50 nmol/L), or sufficient (>50 nmol/L). Demographic variables, documented clinical indications, and comorbidities, including diabetes mellitus, hypertension, anemia, chronic kidney disease, and hypothyroidism, were analyzed, alongside temporal trends in testing patterns.

Results

A total of 3,081 patients were included, predominantly women and Omani nationals. Overall, vitamin D sufficiency was high across the cohort. However, vitamin D insufficiency was more prevalent among younger and middle-aged adults, particularly women, whereas older adults demonstrated higher sufficiency rates. Pediatric vitamin D deficiency was uncommon, though adolescents showed increased rates of insufficiency. A substantial proportion of vitamin D test requests were not aligned with guideline-supported clinical indications. Among individuals with low vitamin D levels, anemia, hypertension, and hypothyroidism were the most frequently observed comorbidities, while diabetes mellitus did not demonstrate a strong association.

Conclusion

Despite increasing testing volumes, clinically significant vitamin D deficiency was relatively uncommon, suggesting the potential overutilization of testing. These findings highlight the need for targeted, indication-based vitamin D testing strategies and strengthened laboratory stewardship to improve clinical value and optimize healthcare resource utilization.

## Introduction

Historically, vitamin D assays were primarily ordered for assessing bone metabolism in conditions such as osteomalacia and rickets [1]. However, the understanding of vitamin D’s broader physiological roles, including its influence on immune function and chronic disease prevention, has expanded significantly, leading to a re-evaluation of its clinical utility beyond these traditional indications [2]. In the past decade, there has been a marked increase in laboratory requests for serum 25-hydroxyvitamin D (25(OH)D) testing, propelled by elevated awareness among the public and clinicians, alongside expanding assertions of its extraskeletal benefits [1]. A noticeable surge in the prescription of vitamin D assays has been observed, with an increase from 9,620 tests in 2008 to 81,641 in 2013, predominantly for 25-hydroxyvitamin D (25(OH)D) [1]. This escalation in testing volume occurred primarily among general practitioners [1]. However, despite this heightened testing frequency, a substantial proportion of these tests, approximately 56%, lack a clear clinical indication, suggesting potential inefficiencies in current screening practices [3].

Moreover, prominent clinical guidelines advise against routine screening for vitamin D deficiency in the general population due to a lack of strong evidence from clinical outcome studies, recommending instead that testing be limited to patients with established risk factors or specific clinical indications [4]. The US Preventive Services Task Force has concluded that current evidence is insufficient to assess the benefits and harms of screening asymptomatic adults, underscoring uncertainty as to whether identifying “low” vitamin D levels results in meaningful health improvements [5]. Likewise, the Choosing Wisely initiative recommends against routine vitamin D testing in low-risk populations, advocating instead for targeted screening in scenarios where test results are anticipated to influence patient management [6]. Recent Endocrine Society guidance also suggests against routine 25(OH)D testing in the absence of established indications [4]. Despite these recommendations, the incidence of vitamin D testing has risen substantially, suggesting a disconnect between clinical guidelines and actual practice [7].

This study initially aimed to determine the prevalence of vitamin D deficiency and insufficiency among both Omani and non-Omani residents, to analyze temporal trends in vitamin D status between 2018 and 2022, and to identify demographic groups at increased risk. During data review, it became evident that a substantial proportion of vitamin D test requests were not aligned with established clinical indications. This observation prompted a broader evaluation of test utilization and clinical justification. Consequently, the study was expanded to assess the association between vitamin D testing and common comorbid conditions, including diabetes mellitus, hypertension, anemia, chronic kidney disease, and hypothyroidism. In addition, the clinical utility of vitamin D testing over the five-year period was evaluated by examining whether test results led to meaningful clinical interpretation or intervention within the same study population. This extended analysis provides insight into both the epidemiology of vitamin D status and the appropriateness of test utilization in routine clinical practice.

## Materials and methods

Study design and setting

This study is part of a broader research program examining vitamin D status and testing practices in primary care health institutes in the region. A retrospective cross-sectional design was employed, utilizing routinely collected clinical and laboratory data. Physicians requested the investigation at primary care institutes in the North Batinah Governorate and performed it at Sohar Hospital, which functions as the primary referral center for the governorate. This hospital also houses the sole laboratory conducting serum 25-hydroxyvitamin D testing for the entire region, ensuring uniform analytical methods, minimizing inter-laboratory variability, and providing a comprehensive and representative regional dataset.

The study period extended from January 2018 to November 2022, enabling a five-year assessment of vitamin D testing patterns and population vitamin D status. The cross-sectional approach allowed for the evaluation of overall prevalence while facilitating subgroup analyses according to demographic characteristics, clinical comorbidities, and test-ordering indications, thereby supporting both epidemiological and utilization-focused objectives of the broader research.

Study population

The study population included all adult Omani and non-Omani residents who underwent serum vitamin D testing during the specified period. Eligibility criteria necessitated patients of any age and gender residing within the region, with a documented serum vitamin D test conducted at the Sohar Hospital laboratory. Conversely, exclusion criteria comprised incomplete records lacking demographic or biochemical data. Following the application of these criteria, the definitive dataset encompassed 3,081 patients. This substantial sample size, coupled with diverse age groups, genders, and nationalities, furnished adequate statistical power for comprehensive subgroup comparisons.

Data collection

Data were systematically extracted from the Al-Shifa electronic medical records system, a nationwide platform in Oman for patient data management. The comprehensive dataset included several categories of information: demographic variables such as age, gender, nationality, and residency; biochemical test results, specifically serum vitamin D levels; and a detailed record of comorbidities, encompassing clinical history of diabetes mellitus, hypertension, anemia, chronic kidney disease, hypothyroidism, and prior bariatric surgery. Additionally, the documented reasons for requesting vitamin D testing were collected, covering clinical indications such as musculoskeletal complaints, dermatological concerns, fatigue, systemic illnesses, or follow-up investigations. Where available, documentation regarding vitamin D supplement intake was also recorded. Initially, the extracted data were exported to Microsoft Excel (Microsoft Corp., Redmond, WA) spreadsheets for cleaning and preliminary organization before being transferred into IBM SPSS Statistics version 20.0 (IBM Corp., Armonk, NY) for subsequent statistical analysis.

Laboratory analysis and vitamin D definitions

Serum 25-hydroxyvitamin D (25(OH)D) levels were determined at the Sohar Hospital laboratory using a chemiluminescent immunoassay, a method established as standard practice across Oman. Consistent results were ensured through strict adherence to national laboratory guidelines for calibration and quality control procedures.

Deficiency was defined as serum 25(OH)D levels below 30 nmol/L, insufficiency as 30-50 nmol/L, and sufficiency as 50 nmol/L or higher [8]. These established thresholds are widely employed in both epidemiological research and clinical settings, thereby enabling comparative analysis with global literature and facilitating their practical implementation in local clinical guidelines [9].

Statistical analysis

Quantitative variables, such as age and serum vitamin D, were presented as means with corresponding standard deviations, while categorical variables, including gender and comorbidity status, were articulated through frequencies and percentages. Temporal trends were ascertained by segmenting the data according to the year of testing and subsequently examining the annual proportions of deficiency, insufficiency, and sufficiency.

Ethical considerations

Ethical clearance for data acquisition and subsequent analysis was secured from the Regional Ethical Approval Research Committee, aligning with the mandates stipulated by the Ministry of Health, Oman. The aforementioned committee undertook a rigorous review and sanctioned the study protocol prior to the commencement of data extraction. Given the retrospective design of this study and its reliance on de-identified patient data, the requirement for individual informed consent was waived. The confidentiality and anonymity of all collected data were rigorously preserved, with access exclusively restricted to authorized personnel. The Research and Ethical Review and Approval Committee of North Batina Governorate issued approval MoH/CSR/23/27089.

## Results

General characteristics of the study population

The study cohort comprised 3,081 patients who underwent serum vitamin D testing between January 2018 and November 2022. The demographic profile indicated that 2,959 (96%) were Omani nationals and 2,511 (81.5%) were women. The overall mean serum vitamin D level across the cohort was 82.5 (±28.02) nmol/L. A discernible pattern revealed that men consistently exhibited higher vitamin D concentrations compared to women. Furthermore, younger participants presented marginally elevated mean vitamin D levels relative to adults; however, these differences were not pronounced. Notably, nationality did not exert a significant influence on vitamin D status within the study population (Table 1).

**Table 1:** Baseline Characteristics and Mean Serum 25-Hydroxyvitamin D Levels of the Study Population SD: standard deviation

Characteristic	Total Number, N (%)	Mean Vitamin D (±SD), nmol/L
Total population	3,081	82.5 (±28.02)
Omani	2,959 (96%)	82.5 (±27.96)
Non-Omani	122 (4%)	80.8 (±29.63)
Male	570 (18.5%)	88.9 (±30.78)
Female	2,511 (81.5%)	81.0 (±27.15)
≥18 years (male)	461 (80.9%)	88.5 (±30.67)
<18 years (male)	109 (19.1%)	90.7 (±31.28)
≥18 years (female)	2,365 (94.2%)	80.9 (±26.65)
<18 years (female)	146 (5.8%)	81.7 (±34.32)
Normal vitamin D	2,720 (88.3%)	79.9 (±27.3)
Insufficient vitamin D	344 (11.2%)	42.9 (±5.25)
Deficient vitamin D	17 (0.6%)	23.5 (±6.28)

Age- and sex-specific patterns of vitamin D status: Key findings

Across the study population, vitamin D sufficiency predominated. In the pediatric population, vitamin D deficiency was uncommon, remaining below 2% across those aged less than 18 years. Adults demonstrated consistently high sufficiency rates, with 2,493 individuals (88.2%), across all age and sex categories. Despite this overall favorable status, distinct demographic variations were observed. Young adult women exhibited the highest prevalence of vitamin D insufficiency, with 147 individuals (14.9%), thus identifying this group as a key at-risk cohort. In contrast, women aged 58 years or older showed significantly higher sufficiency rates, suggesting improved status with advancing age. A similar age-related trend was evident among men, with vitamin D sufficiency progressively increasing across age groups and peaking at 97.4% (n=76) in those aged 58-78 years (Table 2).

**Table 2:** Distribution of Vitamin D Status in the Studied Population

Gender	Age Group (Years)	Deficient Vitamin D, N (%)	Insufficient Vitamin D, N (%)	Normal Vitamin D, N (%)
Both	<18	2 (0.8%)	26 (10.2%)	227 (89.0%)
Male	18-38	2 (1.2%)	16 (9.2%)	155 (89.6%)
Male	38-58	3 (1.5%)	16 (8.2%)	175 (90.3%)
Male	58-78	0	2 (2.6%)	76 (97.4%)
Male	>78	0	1 (6.3%)	15 (93.7%)
Female	18-38	4 (0.4%)	147 (14.9%)	838 (84.7%)
Female	38-58	5 (0.5%)	112 (11%)	901 (88.5%)
Female	58-78	1 (0.3%)	22 (6.8%)	299 (92.9%)
Female	>78	0	2 (5.6%)	34 (94.4%)
Total	All	17 (0.6%)	344 (11.2%)	2,720 (88.3%)

Clinical conditions associated with low vitamin D

In individuals with serum vitamin D concentrations below 50 nmol/L, anemia, hypertension, and hypothyroidism were identified as the predominant comorbidities. Although not statistically significant, men demonstrated marginally elevated prevalence rates of anemia, diabetes, and hypertension compared to women (Table 3).

**Table 3:** Comorbidities Associated With Low Serum Vitamin D Levels CKD: chronic kidney disease

Coexisting Medical Conditions	Total, N (%)	Men, N (%)	Women, N (%)
Anemia	77 (22.3%)	12 (15.6%)	65 (84.4%)
CKD	9 (2.6%)	0	9 (100%)
Diabetes	33 (9.5%)	5 (15.1%)	28 (84.9%)
Hypertension	43 (12.4%)	8 (18.6%)	35 (81.4%)
Bariatric surgery	7 (2.0%)	0	7 (100%)
Hypothyroidism	42 (12.1%)	6 (14.3%)	36 (85.7%)
No documented comorbidities	135 (39.0%)	21 (15.6%)	114 (84.4%)
Total	346 (100%)	52 (15.0%)	294 (85.0%)

Clinical reasons for requesting vitamin D testing

The most frequently documented clinical indications for vitamin D testing were musculoskeletal pain, accounting for approximately one-quarter of requests, followed by general health assessments and dermatological conditions. Non-specific pain and symptoms, including generalized body aches and fatigue, represented a further proportion of requests. Neurological disorders and anxiety or psychiatric conditions were less commonly cited, each contributing a small percentage of test requests. Notably, a small proportion of requests were submitted without any documented clinical information. Systemic comorbidities such as hypertension, diabetes, anemia, and inflammatory disorders collectively constituted a minor fraction of indications. Rare indications included end-stage renal disease on dialysis, headache, constipation, chronic or acute infections, hypocalcemia, and immobility (Table 4).

**Table 4:** Clinical Information Written in the Request for Patients With Vitamin D Level at Insufficient Range ESRD: end-stage renal disease

Clinical Information in Requests	Total Number (%)
Musculoskeletal pain	89 (25.72%)
General examination	72 (20.81%)
Dermatological condition	67 (19.36%)
Non-specific pain and symptoms	46 (13.29%)
Neurological disorders	13 (3.76%)
Anxiety and/or psychiatric disorders	11 (3.18%)
No clinical information	11 (3.18%)
Hypertension	9 (2.60%)
Diabetes: general investigation	7 (2.02%)
Anemia	5 (1.45%)
Inflammatory disorders	4 (1.16%)
ESRD on dialysis	3 (0.87%)
Headache	3 (0.87%)
Constipation	2 (0.58%)
Chronic infection	2 (0.58%)
Hypocalcemia	1 (0.29%)
Immobility	1 (0.29%)
Total	346 (100%)

## Discussion

In this study, individuals with serum vitamin D concentrations below 50 nmol/L were evaluated, alongside associated comorbidities, including anemia, hypertension, and hypothyroidism. Overall, these findings are consistent with prior research demonstrating an association between low vitamin D status and a higher prevalence of chronic conditions [10]. Importantly, within the broader context of this study, clinically significant vitamin D deficiency remained relatively uncommon despite substantial testing, suggesting a potential mismatch between testing practices and clinical necessity.

Anemia and low vitamin D

Vitamin D plays a recognized role in hematopoiesis by modulating erythropoietin responsiveness and suppressing pro-inflammatory cytokines. Consequently, deficiency has been linked to anemia of chronic disease [11]. In the present cohort, the high prevalence of anemia may also reflect the demographic composition, particularly the predominance of women of reproductive age. This group is inherently more susceptible due to physiological factors such as menstrual blood loss and other gynecological influences [12]. These findings align with global data identifying anemia as a significant health burden among women of reproductive age [12]. Therefore, the observed association likely reflects both biological mechanisms and population-specific characteristics.

Hypertension and low vitamin D

The relationship between vitamin D insufficiency and hypertension observed in this study is in agreement with previous reports [13]. Vitamin D contributes to vascular health through its regulatory effects on endothelial function, the renin-angiotensin-aldosterone system, and inflammatory pathways [14]. However, given the multifactorial nature of hypertension, vitamin D deficiency alone is unlikely to fully account for its prevalence in this cohort. Supporting this, studies from similar regional populations indicate that while deficiency is common, its direct contribution to hypertension risk may be limited, suggesting an associative rather than causal relationship [15].

Hypothyroidism and low vitamin D

The association between reduced vitamin D levels and hypothyroidism may be explained by underlying autoimmune mechanisms. Vitamin D insufficiency has been implicated in increased susceptibility to autoimmune thyroid disorders, particularly Hashimoto’s thyroiditis, through impaired immune regulation [16]. While this relationship is biologically plausible, it remains complex and requires confirmation through well-designed prospective studies to establish causality [17].

Highlighted population findings

Beyond comorbidities, notable demographic patterns were observed. The mean age of patients with low vitamin D levels was 37 years, indicating a higher prevalence of insufficiency and deficiency among younger and middle-aged adults compared to older individuals. This finding contrasts with traditional assumptions that older populations are at greater risk due to reduced cutaneous synthesis and dietary intake [18]. In the Omani context, this pattern may reflect more consistent clinical monitoring and supplementation among older adults, whereas younger individuals, particularly women of reproductive age, may be less systematically screened [19]. Additionally, lifestyle factors such as reduced outdoor activity and increased indoor living may contribute to suboptimal vitamin D status in younger populations [20].

Contrary to expectations, diabetes mellitus did not emerge as a prominent associated condition in this cohort, despite its high prevalence in Oman and its known association with other chronic diseases such as hypertension [21,22]. This divergence from anticipated patterns suggests that population-specific factors, including genetic or environmental influences, may modify the relationship between vitamin D status and glycemic control, warranting further investigation.

Test indications and their appropriateness

The findings indicate that vitamin D testing was frequently requested for indications not supported by established clinical guidelines, including non-specific symptoms such as generalized pain and fatigue [23]. While some of these presentations may overlap with conditions affecting bone metabolism [8], especially in regions with a high prevalence of deficiency [24], routine screening in asymptomatic or low-risk individuals is generally not recommended [5]. The high proportion of tests requested for general health assessments and follow-up purposes reflects an increasing tendency to incorporate vitamin D evaluation into routine clinical practice, consistent with global trends [7,25].

This growing demand is further influenced by heightened public awareness, particularly among younger individuals and women, driven by perceived health benefits and increased access to health information [3,26,27]. However, the absence of standardized national guidelines in Oman raises concerns regarding potential over-testing, the misinterpretation of results, and unnecessary healthcare expenditure [18]. The influence of direct-to-consumer testing and health-related media further contributes to this trend, often encouraging testing outside evidence-based recommendations [28].

Moreover, the wide range of clinical indications, including neurological, psychiatric, and systemic conditions, highlights the expanding perception of vitamin D as a multisystem biomarker [29]. Despite this, evidence suggests that a substantial proportion of such testing may be unnecessary and that demand-management strategies can effectively reduce test volumes without compromising patient care [6]. Studies have also shown that clinicians frequently override decision-support systems for non-guideline-supported reasons, reinforcing the need for clearer policies and better adherence to evidence-based practices [23]. Similar trends have been documented internationally, where increased testing has led to significant cost implications without corresponding clinical benefit [6].

Collectively, these findings underscore the need for more targeted, indication-based testing strategies and the development of context-specific guidelines to optimize resource utilization and improve clinical value [30].

Strengths and limitations

This study has several notable strengths, foremost among them a large sample size and the use of a single, centralized laboratory employing uniform analytical methods, thereby minimizing inter-assay and inter-laboratory variability and ensuring consistent testing practices. The extended five-year study period enabled the robust evaluation of the patterns in vitamin D testing and status.

Nevertheless, certain limitations should be acknowledged. The retrospective cross-sectional design limits the ability to establish causal relationships or associations between vitamin D status and associated comorbidities. Furthermore, the incomplete availability of key confounding variables, such as sun exposure, dietary intake, body mass index, and compliance with vitamin D supplementation, may have introduced residual confounding. In addition, reliance on electronic medical records for comorbidity data is subject to documentation variability and potential incompleteness, which may have influenced the accuracy of clinical correlations. As this is a single-center study, the findings may not fully represent the national epidemiology, although the study population accounts for a substantial proportion of the regional population.

## Conclusions

This five-year analysis of vitamin D status and test utilization highlights a notable discrepancy between testing practices and clinical necessity. Despite a substantial increase in vitamin D testing, the prevalence of clinically significant deficiency was low, suggesting that a considerable proportion of testing may be unnecessary. Anemia, hypertension, and hypothyroidism were the most frequently associated comorbidities; however, these relationships appear to be influenced more by demographic and population-specific factors than by direct causation. Notably, younger and middle-aged adults, particularly women, demonstrated higher rates of insufficiency, challenging the conventional assumption that older populations are at greatest risk.

At the same time, the frequent use of vitamin D testing for non-specific or non-guideline-supported indications reflects a broader trend of overutilization, likely driven by increased awareness, clinical uncertainty, and the absence of standardized national guidelines. These findings underscore the need for more targeted, evidence-based approaches to vitamin D testing, with greater emphasis on appropriate clinical indications. Implementing clear guidelines and strengthening laboratory stewardship strategies may help reduce unnecessary testing, optimize resource utilization, and enhance the overall clinical value of vitamin D assessment in routine practice.
